# Analysis of the Oxidative Stress Regulon Identifies *soxS* as a Genetic Target for Resistance Reversal in Multidrug-Resistant Klebsiella pneumoniae

**DOI:** 10.1128/mBio.00867-21

**Published:** 2021-06-08

**Authors:** João Anes, Katherine Dever, Athmanya Eshwar, Scott Nguyen, Yu Cao, Sathesh K. Sivasankaran, Sandra Sakalauskaitė, Angelika Lehner, Stéphanie Devineau, Rimantas Daugelavičius, Roger Stephan, Séamus Fanning, Shabarinath Srikumar

**Affiliations:** a UCD-Centre for Food Safety, UCD School of Public Health, Physiotherapy and Sports Science, University College Dublin, Dublin, Ireland; b Department of Food, Nutrition, and Health, College of Food and Agriculture, United Arab Emirates University, Al Ain, UAE; c Institute for Food Safety and Hygiene, University of Zurich, Zurich, Switzerland; d Genome Informatics Facility, Iowa State University, Ames, Iowa, USA; e Department of Biochemistry, Faculty of Natural Sciences, Vytautas Magnus University, Kaunas, Lithuania; f Université de Paris, BFA, UMR 8251, CNRS, Paris, France; g Institute for Global Food Security, Queen’s University Belfast, Belfast, United Kingdom; McMaster; Indiana University Bloomington

**Keywords:** AMR, *Klebsiella pneumoniae*, mechanisms of resistance, oxidative stress, *soxS*

## Abstract

In bacteria, the defense system deployed to counter oxidative stress is orchestrated by three transcriptional factors, SoxS, SoxR, and OxyR. Although the regulon that these factors control is known in many bacteria, similar data are not available for Klebsiella pneumoniae. To address this data gap, oxidative stress was artificially induced in K. pneumoniae MGH78578 using paraquat and the corresponding oxidative stress regulon recorded using transcriptome sequencing (RNA-seq). The *soxS* gene was significantly induced during oxidative stress, and a knockout mutant was constructed to explore its functionality. The wild type and mutant were grown in the presence of paraquat and subjected to RNA-seq to elucidate the *soxS* regulon in K. pneumoniae MGH78578. Genes that are commonly regulated both in the oxidative stress and *soxS* regulons were identified and denoted as the oxidative SoxS regulon; these included a group of genes specifically regulated by SoxS. Efflux pump-encoding genes and global regulators were identified as part of this regulon. Consequently, the isogenic *soxS* mutant was found to exhibit a reduction in the minimum bactericidal concentration against tetracycline compared to that of the wild type. Impaired efflux activity, allowing tetracycline to be accumulated in the cytoplasm to bactericidal levels, was further evaluated using a tetraphenylphosphonium (TPP^+^) accumulation assay. The *soxS* mutant was also susceptible to tetracycline *in vivo* in a zebrafish embryo model. We conclude that the *soxS* gene could be considered a genetic target against which an inhibitor could be developed and used in combinatorial therapy to combat infections associated with multidrug-resistant K. pneumoniae.

## INTRODUCTION

Oxygen started accumulating in the biosphere about 2 to 3 billion years ago. Many organisms harvest energy by oxidizing organic compounds, with oxygen acting as the terminal electron acceptor. This molecule, therefore, has become essential for life, at least for aerobic organisms. As a natural consequence of aerobic metabolism, the production of toxic reactive oxygen species (ROS), namely, hydrogen peroxide (H_2_O_2_), superoxide radical (O_2_^·−^), and the generation of hydroxyl radical (HO^·^), is inevitable in an oxygen-rich environment. Different ROS will not only oxidize macromolecules (such as DNA, proteins, and lipids) but also extract iron from proteins containing iron-sulfur clusters, creating a highly reactive HO^·^-rich intracellular environment (1, 2) detrimental for bacteria. Therefore, to survive the effects of ROS, bacteria deploy a variety of adaptive responses. These are well-characterized in bacteria like Escherichia coli (3) but, as yet, not in Klebsiella pneumoniae.

In bacteria, the primary antioxidant defense systems employ superoxide dismutase (SOD) and catalase (CAT) enzymes (1, 4). However, these may prove inadequate to protect bacteria under circumstances of extreme and prolonged oxidative stress. Under these stress conditions, bacteria can activate the OxyR and SoxRS systems in response to hydrogen peroxide (5) and redox-active compounds (6), respectively. Both OxyR and SoxRS work by transcriptionally activating genes whose protein products function either to protect or repair damage caused by intracellular ROS accumulation. In the SoxRS system, the activation of a target gene occurs via a two-step process wherein SoxR acts as a sensory protein recognizing elevated levels of ROS. Under normal conditions (nonstressed), the binuclear iron-sulfur clusters [2Fe-2S] in the SoxR protein remain reduced. In the presence of enhanced levels of superoxides, the [2Fe-2S] clusters are oxidized (7). Oxidization of the SoxR protein enhances an open complex formation with RNA polymerase, thereby activating transcription of *soxS* (8). The SoxS protein is a transcriptional activator belonging to the XylS/AraC family (7). SoxR-dependent induction of SoxS in turn activates the transcription of many other genes (denoted collectively as the SoxRS regulon) whose primary functions involve antioxidative action, detoxification, efflux of redox-active compounds, changes in membrane permeability, and protecting DNA, thereby rescuing bacteria from the deleterious effects of increased intracellular levels of ROS (9–11). Overall, the biological role of the SoxRS regulon can be summarized as (i) prevention of oxidative damage, (ii) recycling of damaged macromolecules, and (iii) regeneration of NADP.

In E. coli, genes that were regulated by SoxS were identified (12–17). Although the transcriptional organization of *soxS* is well characterized in some pathogens, the data is lacking for K. pneumoniae. These bacteria are a member of the ESKAPE group, one of six pathogens responsible for most drug-resistant nosocomial infections (18). Since oxidative stress is known to mediate antibiotic resistance in pathogens, we were interested in identifying how K. pneumoniae responds to oxidative stress and what its impact might be on antimicrobial resistance.

In this study, transcriptome sequencing (RNA-seq) was used to describe the transcriptional architecture of K. pneumoniae MGH78578 during exposure to a reactive oxygen species (ROS)-inducing agent, paraquat, revealing that the regulon was controlled by the *soxRS* two-component system. RNA-seq analysis of the K. pneumoniae MGH78578 Δ*soxS* isogenic mutant was carried out and used to describe the oxidative *soxS* regulon, a stringent set of genes regulated via *soxS.* K. pneumoniae MGH78578 Δ*soxS* was found to be highly susceptible to tetracycline. Susceptibility of the mutant to tetracycline coupled with increased accumulation of tetraphenylphosphonium (TPP^+^) in the bacterial cytoplasm was supported at least in part by the downregulation of *acrAB-tolC* and the global regulator *marRAB* in K. pneumoniae MGH78578 Δ*soxS.* Since the mutant was highly avirulent in a zebrafish model, we predict that *soxS* can be used as a genetic target to inhibit infections associated with multidrug-resistant (MDR) K. pneumoniae.

## RESULTS AND DISCUSSION

### SoxS is the major transcriptional regulator when K. pneumoniae MGH78578 is exposed to redox compound-based oxidative stress.

Experimentally, oxidative stress can be induced in bacteria by exposing cultures to either redox compounds like PQ (paraquat) or H_2_O_2_. PQ is 1,1-dimethyl-4,4-bipyridinium and is a widely used nonselective herbicide, found to induce oxidative stress by enhancing ROS levels, superoxide anion radical (^·^O_2_^−^), in a dose-dependent manner, as exemplified in Vibrio cholerae, E. coli, and others (14, 19). First, we started by assessing the inhibitory concentration of PQ in K. pneumoniae MGH78578 using broth microdilution and determined the MIC to be 15.62 μM. Thereafter, the following transcriptomic experiments were carried out at sub-MICs (half the MIC). Here, we used RNA-seq to investigate the genome-wide transcriptional architecture of multidrug-resistant K. pneumoniae MGH78578 following exposure to a subinhibitory concentration of PQ. K. pneumoniae MGH78578 was exposed to 7.8 μM PQ for 30 min to induce oxidative stress. These PQ-induced cultures (denoted MGH_PQ_ A and B) along with a parallel set of unexposed wild-type (WT) bacterial cells (denoted MGH_wt_ A and B) were subjected to RNA isolation and deep-level sequencing (see Table S1, WS1, in the supplemental material). As discussed in detail later, the *soxS* gene, an XylS/AraC-type transcriptional regulator of oxidative stress, was one of the most highly induced genes during oxidative stress. Therefore, to understand the *soxS*-mediated oxidative stress, we constructed the K. pneumoniae MGH78578 Δ*soxS* mutant and carried out RNA-seq on the deletion strain grown in the presence of PQ (denoted MGH*ΔsoxS*_PQ_ A and B libraries). Altogether, six RNA-seq libraries were generated in this study.

10.1128/mBio.00867-21.3 TABLE S1 (WS1) RNA-seq mapping details. This table provides details of the RNA-seq reads mapped against different regions of the K. pneumoniae MGH78578 genome are. A complete list of all primers used in the experiments is also provided. (WS2) The table presents data describing gene expression patterns of Klebsiella pneumoniae MGH75878 (MGH_PQ_ versus MGH_WT_) and K. pneumoniae MGH75878 Δ*soxS* (MGH *Δsox*S_PQ_ versus MGH_PQ_) during oxidative stress. Column A shows a new gene ID, and column C shows the original name, while column B represents the gene name. Columns X/Y/Z/AA/AB/AC/AD shows raw reads generated from RNA-seq conducted on different libraries, while columns AF/AG/AH/AI/AJ/AK represents the normalized reads. The fold changes were calculated from the normalized reads obtained from different libraries, MGH_PQ_ versus MGH_WT_ (column K) and MGH Δs*oxS*_PQ_ versus MGH_PQ_ (column U). The genes with statistically significant data obtained from two biological replicates are indicated by a mark (√) in columns J and T based on the *P* value indicated in columns M and W. Based on the differential expression indicated in columns K and U (for statistically significant genes), the differential expression of each gene is indicated by a color code in columns E/F/G/H and O/P/Q/R. Genes highlighted in red indicate antimicrobial resistance genes, while those highlighted in blue indicate genes associated with virulence. (WS3) The table shows the oxidative *soxS* regulon of Klebsiella pneumoniae MGH78578, consisting of a stringent set of genes that are regulated by SoxS. The gene list was obtained from statistically significant genes from oxidative regulon and *soxS* regulon with a distinct expression pattern. Genes that were upregulated in the oxidative regulon plus downregulated in the *soxS* regulon and downregulated in oxidative regulon plus upregulated in the *soxS* regulon are shown. Download Table S1, XLSX file, 2.3 MB. Copyright © 2021 Anes et al. 2021 Anes et al. https://creativecommons.org/licenses/by/4.0/ This content is distributed under the terms of the Creative Commons Attribution 4.0 International license.

Approximately 57 million uniquely mapped reads were generated across all six libraries, accounting for more than 9 million reads/library (Table S1, WS1), data that was sufficient for robust transcriptional analysis (20). The expression levels of 5,185 K. pneumoniae MGH78578 chromosomal genes and the resident plasmid carrying genes (including plasmids pKPN3, pKPN4, pKPN5, pKPN6, and pKPN7) were calculated using the *Voom* approach (limma package) (21). We confirmed the reproducibility of the RNA-seq data by calculating the Spearman coefficients for the biological replicates of all libraries based on the normalized read counts. In all six libraries, the coefficient was found to be ∼0.96 to 0.99, confirming the statistical significance between replicates (Fig. S1).

10.1128/mBio.00867-21.1 FIG S1 Graphs showing the distribution of RNA-seq reads in each dataset. Download FIG S1, PDF file, 0.05 MB. Copyright © 2021 Anes et al. 2021 Anes et al. https://creativecommons.org/licenses/by/4.0/ This content is distributed under the terms of the Creative Commons Attribution 4.0 International license.

**FIG 1:**
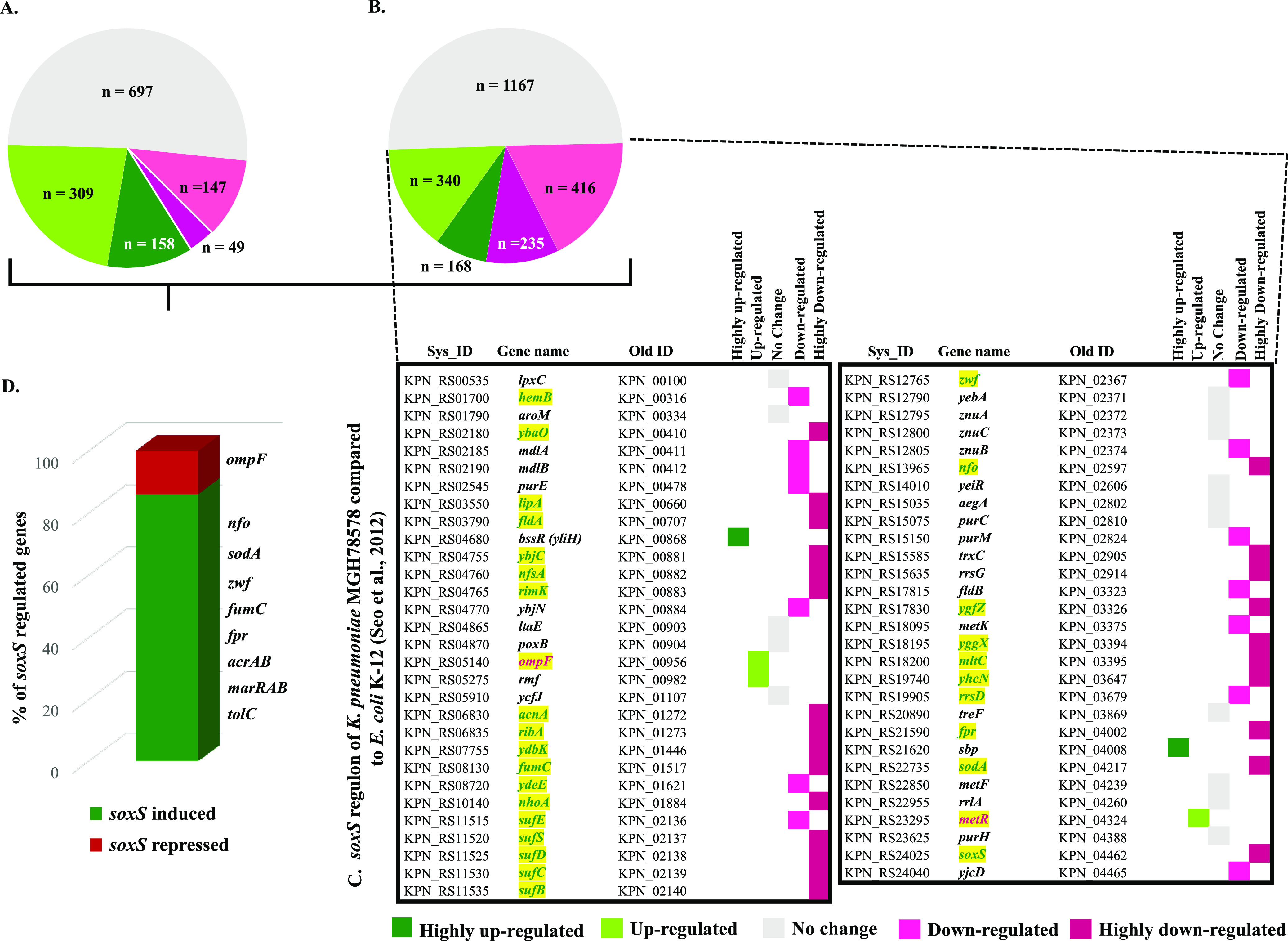
Oxidative, *soxS*, and oxidative *soxS* regulon of K. pneumoniae MGH78578. (A) The number of statistically significant genes identified in the oxidative regulon of K. pneumoniae MGH78578. These are categorized according to their expression pattern and depicted in a color code based on the color key given below. (B) The number of statistically significant genes identified in the *soxS* regulon and categorized according to their expression pattern. (C) Differentially regulated genes common in the *soxS* regulon of both K. pneumoniae MGH78578 and E. coli K-12 (3). The genes that are in green font and highlighted in yellow are those identified in the oxidative *soxS* regulon. (D) The number of genes in the oxidative *soxS* regulon of K. pneumoniae MGH78578 expressed as a percentage. The most significant *soxS*-induced and -repressed genes are indicated.

Here, we describe the oxidative stress regulon of K. pneumoniae MGH78578 by identifying the genes that were differentially regulated in MGH_PQ_ versus MGH_wt_ libraries. The oxidative stress regulon was comprised of 1,366 genes that were differentially regulated (Fig. 1A and Table S1, WS2). Of these, 11.5% (*n* = 158) were highly upregulated (>4-fold) and 22.5% (*n* = 309) were upregulated (2- to 4-fold). A total of 49 genes (3.7%) were highly downregulated (>4-fold), while a further 147 (11.12%) were downregulated (2- to 4-fold) (Table S1, WS2). Upon analysis, the most induced K. pneumoniae MGH78578 gene was found to be *soxS* (145-fold), indicating that the *soxRS* regulon was highly active in PQ-exposed K. pneumoniae MGH78578. The transcriptomic response of bacteria to oxidative stress is specific to the agent causing oxidative stress; extracellular H_2_O_2_ triggers the OxyR regulon, while PQ induces the SoxRS regulon, as exemplified in Escherichia coli (3).

**FIG 2:**
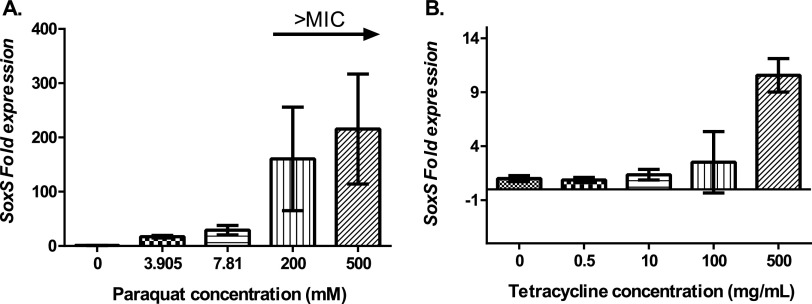
Expression of *soxS* gene under paraquat stress (A) and tetracycline stress (B). In both cases, the compounds were added to bacteria growing at mid-exponential phase for 30 min. Error bars represent standard deviations (SD) calculated from three biological replicates with three technical replicates each.

Based on these E. coli data, we hypothesized that exposure to PQ should induce the SoxS regulon in K. pneumoniae MGH78578. Since no data were available in K. pneumoniae, we put our hypothesis to the test using reverse transcription-quantitative PCR (RT-qPCR) targeting the *soxS* gene. Our RT-qPCR data confirmed that the expression of the *soxS* transcript improved with increasing concentrations of PQ (Fig. 2A).

Exposure to H_2_O_2_, however, generated a different response in other bacteria. Exposure of V. cholerae to oxidative stress increased the activity of SOD and CAT enzymes (19). However, in V. cholerae, the level of CAT did not increase postexposure to PQ but rather increased during exposure to H_2_O_2_. Our results describing PQ-exposed K. pneumoniae MGH78578 support this observation: none of the catalases (encoded by genes KPN_RS06170, KPN_RS06615, and KPN_RS09805) were differentially regulated (Table S1, WS2). However, the SOD (encoded by *sodA*, *sodB*, and *sodC*) was highly upregulated; *sodA* alone was highly upregulated (14-fold), while *sodC* was upregulated (∼3-fold) in PQ-induced cells. We did not find *sodB* to be differentially regulated within PQ-treated K. pneumoniae MGH78578. It is tempting to speculate that selective differential regulation of SOD and not CAT in K. pneumoniae MGH78578 is the response to O2·- induced by PQ.

Since SoxS, an XylS/AraC-type transcriptional regulator, was highly induced following exposure to PQ, we were interested in identifying the associated genes that were differentially regulated. For this, we constructed a K. pneumoniae MGH78578 Δ*soxS* mutant. We cultured the mutant, exposed the cells to PQ, and again used RNA-seq (MGHΔ*soxS*_PQ_ library) to identify the differentially regulated genes (MGHΔ*soxS*_PQ_ library versus MGH_PQ_ library), thereby comprising the *soxS* regulon. The *soxS* regulon was made up of 2,326 differentially regulated genes (Table S1, WS2) (Fig. 1B). Of these, 7.2% of the genes (*n* = 168) were highly upregulated (>4-fold) and 14.6% (*n* = 340) were upregulated (2- to 4-fold). A total of 235 genes (10%) were highly downregulated (>4-fold), while 416 (17.8%) were downregulated (2- to 4-fold) (Table S1, WS2) (Fig. 1B). To demonstrate the robustness of these data, we compared our K. pneumoniae MGH78578 *soxS* regulon with the E. coli *soxS* regulon published earlier (3). Of the 59 *soxS* genes regulated in E. coli K-12, 44 were also found to be similarly regulated by *soxS* in K. pneumoniae MGH78578 (Fig. 1C).

To add stringency to our data, we further compared the oxidative stress regulon to the *soxS* regulon to identify those genes that belonged to the oxidative *soxS* regulon. The oxidative *soxS* regulon represented a stringent set of K. pneumoniae MGH78578 genes that were regulated by *soxS* alone. The genes belonging to this regulon had a characteristic statistically significant expression pattern, upregulated in MGH_PQ_ (*w.r.t.* [with respect to] MGH_wt_) and downregulated in MGHΔ*soxS*_PQ_ (*w.r.t.* MGH_PQ_) (i.e., SoxS induced); downregulated in MGH_PQ_ (*w.r.t.* MGH_wt_); and upregulated in MGHΔ*soxS*_PQ_ (*w.r.t.* MGH_PQ_) (i.e., SoxS repressed). In total, 256 genes belonged to the oxidative *soxS* regulon. Of these, 222 genes were found to be SoxS induced, while 34 were SoxS repressed (Table S1, WS3). Examples include *soxS*, *acrAB*, *tolC*, and *oqxAB*, among others, all of which were *soxS* induced. Of the 44 genes commonly identified in our *soxS* regulon and E. coli K-12 (3), 30 were identified to belong to the more stringent oxidative *soxS* regulon. Our oxidative *soxS* regulon identified many genes that were previously shown to be regulated by SoxS. A discussion of these genes is included in Text S1.

10.1128/mBio.00867-21.2 TEXT S1 A text file describing 44 genes commonly identified in our *soxS* regulon and E. coli K-12 (3), some 30 were identified to belong to the more stringent oxidative *soxS* regulon. Our oxidative *soxS* regulon identified many genes that were previously shown to be regulated by SoxS. Download Text S1, DOCX file, 0.03 MB. Copyright © 2021 Anes et al. 2021 Anes et al. https://creativecommons.org/licenses/by/4.0/ This content is distributed under the terms of the Creative Commons Attribution 4.0 International license.

K. pneumoniae MGH78578, isolated from the sputum of a 66-year-old intensive care unit patient in 1994, is a multidrug-resistant isolate, and its antimicrobial resistance profile is well characterized (22). This strain is resistant to ampicillin, oxacillin, ticarcillin, trimethoprim-sulfamethoxazole, nalidixic acid, kanamycin, gentamicin, and tetracycline but is susceptible to amikacin, ciprofloxacin, and imipenem. Our primary interest was in identifying how *soxS* modulates antimicrobial resistance in K. pneumoniae MGH78578. Thus, we assayed whether any K. pneumoniae MGH78578 genes conferring antimicrobial resistance were captured in our oxidative *soxS* regulon. We identified 11 antimicrobial resistance-encoding genes (*acrAB*, *acrE*, *tolC*, *marRAB*, *oqxAB*, *cmr* [*mdfA*], *ybhT*, KPN_RS15915, and KPN_RS15920) in the oxidative *soxS* regulon, and all of them were *soxS* induced (Table S1, WS3). Interestingly, we also found that another member of the XylS/AraC family, *tetD*, encoding a tetracycline efflux MFS transporter, was identified in the *soxS* regulon and not in the oxidative regulon. This shows that, at least in K. pneumoniae, *tetD* is positively regulated by *soxS*. Although *tetD* was shown to modulate response against redox compounds and tetracycline (23), we did not find any evidence of differential regulation when K. pneumoniae MGH78578 was exposed to PQ. Since many genes conferring antimicrobial resistance were modulated by *soxS*, we were interested in examining whether the inactivation of *soxS* resulted in aberrations in the antimicrobial resistance pattern of K. pneumoniae MGH78578.

### Deletion of *soxS* in multidrug-resistant K. pneumoniae MGH78578 produced a reduction in the minimal bactericidal concentration (MBC) against antimicrobials, particularly tetracycline.

The deletion of a transcriptional regulator like *soxS* could have a large impact on cell metabolism and stress responses. To globally visualize the metabolic aberrations concerning *soxS*, we subjected the K. pneumoniae MGH78578 WT and its isogenic Δ*soxS* mutant to multiple growing conditions on a phenotypic microarray platform. Of the 1,484 conditions tested, altered phenotypes (WT versus mutant) were observed under 517 conditions, of which only 12 were significantly upregulated, wherein the mutant showed increased respiratory metabolism compared with the WT (Table S2). Significant phenotypic alterations were found to be associated with nitrogen and some nitrogen peptides and with amino acid sources such as l-isoleucine, l-ornithine, and glycine. This analysis also identified 50 conditions determined to be downregulated and in which the mutant showed reduced metabolic respiration compared to the WT. These were found to be associated with high pH (5, 9) sensitivity and antimicrobial drugs such as tetracyclines (doxycycline, demeclocycline, chlortetracycline, and minocycline), aminoglycosides (amikacin), cephalosporins (cephalothin, cefuroxime, and cefotaxime), β-lactams (cloxacillin, oxacillin, and phenethicillin), and others, including polymyxin B (PMB) and colistin (polymyxin E).

10.1128/mBio.00867-21.4 TABLE S2 Metabolic activities of K. pneumoniae MGH78578 (wild type) and its isogenic *ΔsoxS* mutant grown under multiple conditions on the OmniLog microarray platform. Significant metabolic changes for delta activities were <−2 and >2. Download Table S2, XLSX file, 0.08 MB. Copyright © 2021 Anes et al. 2021 Anes et al. https://creativecommons.org/licenses/by/4.0/ This content is distributed under the terms of the Creative Commons Attribution 4.0 International license.

Our RNA-seq data showed that the genes encoding antimicrobial resistance, such as *acrAB-tolC*, *marRAB*, and others, were differentially regulated in the K. pneumoniae MGH78578 Δ*soxS* strain and, thus, classified as *soxS* induced. This observation, and the phenotypic microarray associated with metabolic profiling, led us to hypothesize that the *soxS* mutant has a modified antimicrobial resistance profile compared to the wild type. To test our hypothesis, we assayed the MIC/MBC of both K. pneumoniae MGH78578 and K. pneumoniae MGH78578 Δ*soxS* strains against a panel of antimicrobial compounds. MIC/MBC assays were carried out on K. pneumoniae MGH78578 and K. pneumoniae MGH78578 Δ*soxS* strains against colistin, kanamycin, gentamicin, cefotaxime, and tetracycline (Table 1). Escherichia coli ATCC 25922 was used as a control. Our results showed that there was no significant change in the MIC/MBC values between the mutant and wild type against colistin, kanamycin, and rifampin, even though our phenotypic microarray assay recorded downregulation in the metabolism of the mutant compared to the wild type. It could be that the metabolic downregulation was not sufficient to cause an inhibitory effect. However, there was a significant reduction in the MIC/MBC values for the K. pneumoniae MGH78578 Δ*soxS* strain compared to K. pneumoniae MGH78578 when exposed to tetracycline (64- and 8-fold, respectively) and cefotaxime (32- and 64-fold, respectively).

**TABLE 1:** Minimum inhibitory and bactericidal concentrations determined for the wild-type K. pneumoniae MGH78578 and the K. pneumoniae MGH78578 Δ*soxS* isogenic mutant

Antimicrobial compound	MICs and MBCs^*a*^ (μg/ml)			
	K. pneumoniae MGH78578		K. pneumoniae MGH78578 Δ*soxS*	
	MIC	MBC	MIC	MBC
Colistin	0.25 (S)	0.5	0.125 (S)	0.125
Kanamycin	>512 (R)	>512	>512 (R)	>512
Gentamicin	128 (R)	128	64 (R)	64
Cefotaxime	32 (R)	64	1 (S)	1
Tetracycline	128 (−)	128	2 (−)	16

a Results indicate the median value from 3 independent assays. MIC values shown are interpreted according to EUCAST guidelines. (S), susceptible; (R), resistant; (−) not available.

Therefore, using a combination of phenotypic microarray and RNA-seq, we show that tetracycline tolerance was *soxS* dependent in MDR K. pneumoniae MGH78578. Oxidative stress is a common cause of cell death mediated by antimicrobial agents, irrespective of the class to which the compound belongs (24). Therefore, we were interested to know whether exposure to tetracycline induced any oxidative stress in K. pneumoniae MGH78578. For this, we checked the induction of *soxS* in tetracycline-exposed K. pneumoniae MGH78578. Proportional induction of *soxS* expression in response to increasing tetracycline concentration confirmed the exposure to tetracycline-induced *soxS*-dependent oxidative stress in K. pneumoniae MGH78578 (Fig. 2B).

The *soxRS*-associated regulation of antibiotic resistance was described earlier in several bacteria (25, 26). Similarly, the induction of ROS was also reported to modulate antibiotic resistance in other pathogenic bacteria. For example, Salmonella enterica serovar Typhimurium was shown to modulate its susceptibility to tetracycline when exposed to an ROS-generating macrolide antibiotic, tylosin (27). In Acinetobacter baumannii, *soxR* overexpression also led to susceptibility to tetracycline (28). This SoxR-based negative regulation of SoxS could be the reason underpinning the increased susceptibility. Even though the correlation between the expression of *soxS* and efflux pumps has been shown previously (29), there is no evidence pointing to the cytoplasmic accumulation of antimicrobial compounds due to an inactive *soxS*-based impaired efflux activity. We therefore proceeded to determine whether an impaired efflux activity led to the accumulation of compounds within the cytoplasm of the K. pneumoniae MGH78578 Δ*soxS* mutant, leading to the bactericidal effect.

### Reduction in accumulation is due to the impaired efflux pump activity in K. pneumoniae MGH78578 Δ*soxS* cells.

Since the K. pneumoniae MGH78578 Δ*soxS* mutant was susceptible to tetracycline, we were interested in understanding the mechanism underpinning the observation. Our RNA-seq data revealed that the genes encoding the AcrAB-TolC efflux pump were highly SoxS dependent, because they were >4-fold upregulated in the PQ regulon and >8-fold downregulated in the K. pneumoniae MGH78578 Δ*soxS* mutant. Tetracycline is one of several structurally diverse substrates of the efflux pump AcrAB-TolC (30). Hence, we hypothesized that the deletion of the *soxS* gene could lead to a reduction in the expression of the AcrAB-TolC efflux pump. This feature then could account for the accumulation of tetracycline in the cytoplasm to bactericidal levels.

**FIG 3:**
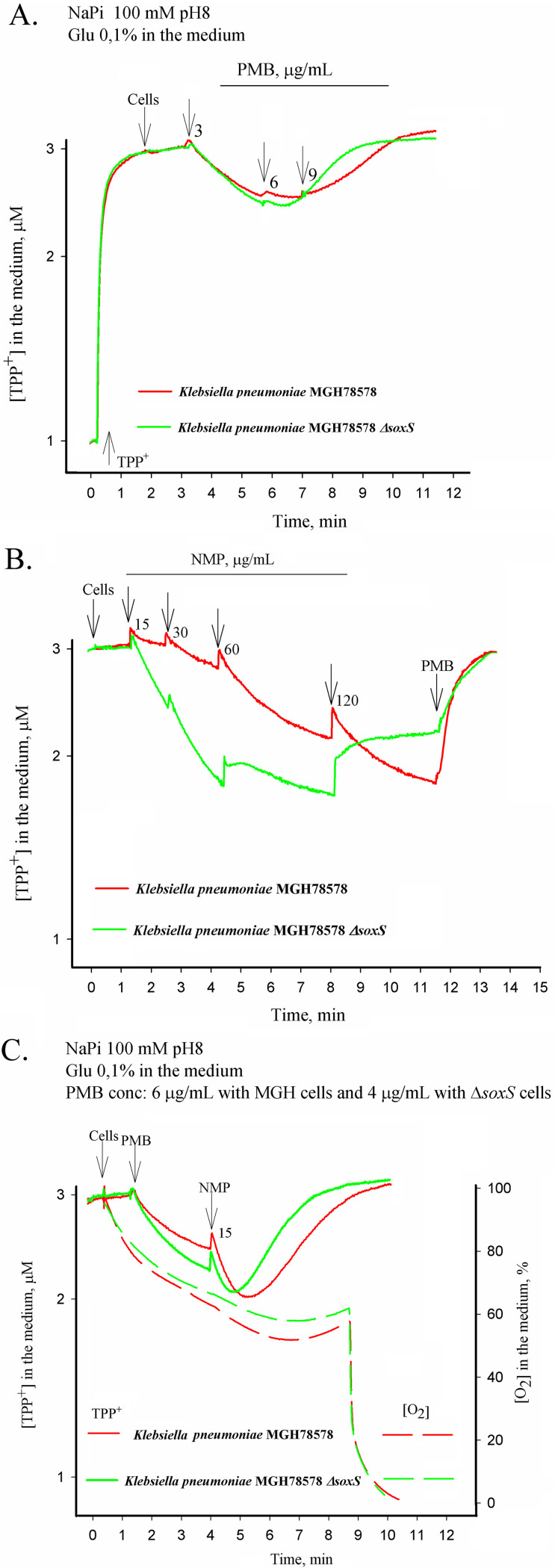
TPP^+^ accumulation in K. pneumoniae MGH78578 and its isogenic mutant MGH78578 Δ*soxS*. All measurements were performed in 100 mM NaPi buffer containing 0.1% glucose, pH 8.0. Concentrated cell suspensions were added to obtain OD_600_ of 1. Final concentrations of NMP (μg/ml) are indicated in panels B and C. The final concentrations of polymixin B (PMB) are indicated in the figure: 3, 6 and 9 μg/ml (A), 50 μg/ml (B), or 6 and 4 μg/ml for *wt* and *ΔsoxS* cells, respectively (C).

To test our hypothesis, we assayed the efflux activity of wild-type K. pneumoniae MGH78578 and K. pneumoniae MGH78578 Δ*soxS* strains by measuring the accumulation of tetraphenylphosphonium (TPP^+^) ions using previously described protocols (31). We first tested whether the K. pneumoniae MGH78578 Δ*soxS* mutant had an intact outer membrane. In this case, both wild-type K. pneumoniae MGH78578 and K. pneumoniae MGH78578 Δ*soxS* strains were first exposed to low concentrations of PMB, an antibiotic that causes outer membrane destabilization, and then assayed the accumulation of TPP^+^. Our results showed that the K. pneumoniae MGH78578 Δ*soxS* strain was more susceptible to PMB, and a concentration of 6 μg/ml was sufficient to induce the depolarization of the plasma membrane. In comparison, for the wild-type K. pneumoniae MGH78578, a concentration of PMB of 9 μg/ml was required. Nonetheless, alterations in membrane voltage (maximum amount of TPP^+^) were similar for both wild-type K. pneumoniae MGH78578 and the isogenic K. pneumoniae MGH78578 Δ*soxS* mutant, showing that neither the outer nor the inner plasma membranes were compromised in the K. pneumoniae MGH78578 Δ*soxS* strain (Fig. 3A). This finding was supported by our earlier RNA-seq data, which showed that membrane-associated genes that were differentially regulated during 1-(1-naphthyl methyl)-piperazine (NMP) (a chemosensitizer) treatment (32) were not differentially regulated in the *soxS* regulon.

Next, we investigated whether the efflux pump activity was compromised in the K. pneumoniae MGH78578 Δ*soxS* strain compared to that of wild-type K. pneumoniae MGH78578. The aim was to confirm/refute our hypothesis that the impaired pump activity could result in the accumulation of tetracycline within the K. pneumoniae MGH78578 Δ*soxS* strain. We previously established that the treatment of K. pneumoniae MGH78578 with NMP destabilized the bacterial outer membrane before efflux pump inhibition and that this phenotype was concentration dependent (32). Hence, we used different concentrations of NMP to test the efflux pump inhibition of K. pneumoniae MGH78578 Δ*soxS* cells compared to that of K. pneumoniae MGH78578. Initially, we treated wild-type K. pneumoniae MGH78578 with NMP and assayed the cells for TPP^+^ accumulation. As expected, in wild-type K. pneumoniae MGH78578, NMP impaired efflux pump activity and induced cytoplasmic TPP^+^ accumulation at a concentration of 30 μg/ml. However, for the K. pneumoniae MGH78578 Δ*soxS* strain, 15 μg/ml NMP was sufficient to inhibit the efflux pump activity and cause TPP^+^ accumulation (Fig. 3B). The increased sensitivity of the K. pneumoniae MGH78578 Δ*soxS* strain to NMP also was observed at 120 μg/ml, where this agent increased the accumulation of TPP^+^ in K. pneumoniae MGH78578 but induced a partial depolarization of the plasma membrane and leakage of the accumulated cation in the K. pneumoniae MGH78578 Δ*soxS* strain. These results show that the efflux pump activity was impaired in the mutant.

To further confirm the effect of the outer membrane destabilization on TPP^+^ accumulation, we pretreated both K. pneumoniae MGH78578 and K. pneumoniae MGH78578 Δ*soxS* strains first with PMB to permeabilize the outer membrane and then retested for NMP-mediated TPP^+^ accumulation. These data indicated that TPP^+^ was accumulated at 15 μg/ml for the K. pneumoniae MGH78578 Δ*soxS* strain, implying that both efflux pump inhibition and outer membrane destabilization could cause TPP^+^ accumulation in the bacterial cytoplasm (Fig. 3C). The respiration activity of the K. pneumoniae MGH78578 Δ*soxS* strain when measured was very close to that of K. pneumoniae MGH78578. Overall, our experiment describing the accumulation of TPP^+^ even at very low concentrations of NMP confirmed that the K. pneumoniae MGH78578 Δ*soxS* strain exhibited an impaired efflux pump activity, and, as the concentration of NMP increased, membrane stability was affected, resulting in TPP^+^ accumulation.

Tetracycline is a substrate for the AcrAB-TolC efflux pump. Taking together the downregulation of *acrAB*-*tolC* and impaired efflux pump activity in the K. pneumoniae MGH78578 Δ*soxS* strain, we conclude that tetracycline accumulates within the cytoplasm of the K. pneumoniae MGH78578 Δ*soxS* strain to bactericidal concentrations, making the mutant susceptible to this antibiotic.

### K. pneumoniae MGH78578 Δ*soxS* strain was avirulent in a zebrafish infection model.

**FIG 4:**
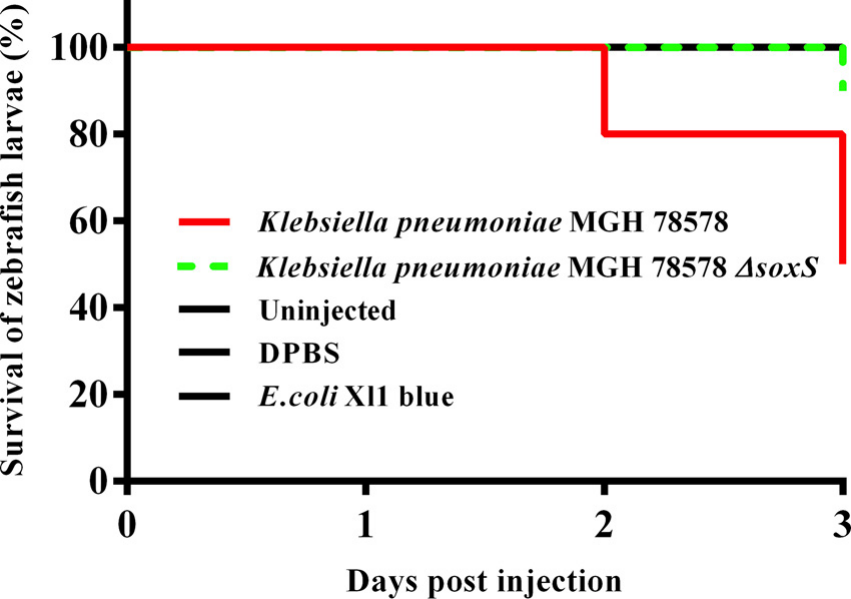
Survival of larvae in a zebrafish infection model. The survival of larvae infected with the isogenic wild-type K. pneumoniae MGH78578 reduced as the days postinfection increased (red line). The survival of larvae infected with the K. pneumoniae MGH78578 Δ*soxS* strain (green line) was maintained at 100% throughout the experiment similar to the negative (avirulent E. coli XI1 blue, uninfected and DPBS) (black line) controls. Log-rank (Mantel-Cox) test, *P* < 0.0001.

Since SoxS mediates oxidative stress, we sought to characterize the role of *soxS* to mitigate oxidative stress in an *in vivo* model. We used a zebrafish (*Danio rero*) embryo model to investigate the survival of the K. pneumoniae MGH78578 Δ*soxS* strain compared with the wild-type K. pneumoniae MGH78578. Zebrafish larvae are generally used as infection models because they are genetically tractable and optically accessible, and they present a fully functional immune system with macrophages and neutrophils that mimic their mammalian counterparts (33). Moreover, a zebrafish larva model was recently used to assay infection associated with K. pneumoniae (34). Wild-type K. pneumoniae MGH78578, K. pneumoniae MGH78578 Δ*soxS*, and E. coli Xl1 blue (an avirulent control) bacterial strains and Dulbecco’s phosphate-buffered saline (DPBS; uninoculated control) were directly injected into the caudal vein of 48 hpf (hours postfertilization) zebrafish embryos and survival rates recorded by observing the presence or absence of a heartbeat postinfection. This time point was selected because the innate immune system begins to develop with primitive macrophages at 24 hpf while neutrophils develop later, at 48 hpf (35). Neutrophils are known to use oxidative stress to control bacterial infections in zebrafish larvae. We observed that the K. pneumoniae MGH78578 Δ*soxS* strain was inefficient in killing zebrafish larvae compared to the wild type (Fig. 4).

At 1 dpi (days postinfection), a survival rate of 100% was recorded in embryos injected with wild-type K. pneumoniae MGH78578, K. pneumoniae MGH78578 Δ*soxS*, and E. coli strains and DPBS. However, at 2 dpi, the survival rate of the embryos injected with wild-type K. pneumoniae MGH78578 decreased to 80%, while the survival rate in those embryos injected with the K. pneumoniae MGH78578 Δ*soxS* strain and in the avirulent/uninoculated controls remained unaltered. At 3 dpi, the survival rate dropped further to 50% for the embryos injected with K. pneumoniae MGH78578 while being maintained at 90% for the K. pneumoniae MGH78578 Δ*soxS* strain-infected embryos (Fig. 4). The survival rate remained unaltered in the case of E. coli- and DPBS-injected embryos over the time course of infection. Recent studies report that high neutrophil recruitment and zebrafish lethality is observed with K. pneumoniae if directly injected into the blood (34, 36). We anticipate two possibilities for the sensitivity of the K. pneumoniae MGH78578 Δ*soxS* strain in zebrafish larvae. First, for vertebrates, extracellular bactericidal action is initiated by neutrophils at a distance by activating the NADPH oxidase-dependent production of superoxide (37). The avirulent phenotype of the K. pneumoniae MGH78578 Δ*soxS* strain could be due to the inefficiency in combating the extracellularly produced neutrophil-originated superoxide in the blood. Second, the K. pneumoniae MGH78578 Δ*soxS* strain exhibited downregulated expression of *acrAB-tolC*, which could result in an avirulent phenotype, as seen previously in Salmonella Typhimurium (38).

### By impairing *soxS*, multidrug-resistant K. pneumoniae MGH78578 infections can be treated by tetracycline.

Currently, various strategies are being investigated to mitigate the threat of AMR in bacteria (39). It was thought that restricting the use of a particular antibiotic would restore susceptibility to that compound over time by eliminating the selective advantage, but it has been observed that AMR is persistent over decades (40). Recent research has elaborated on the possibility wherein resistance can be reversed. One strategy here made use of defined drug-adjuvant combinations to reverse resistance so that conventional antibiotics continue to be effective (41). With this broad goal in mind, we endeavored to identify genetic targets that regulate resistance and develop strategies to reverse resistance by inhibiting them. Our transcriptomic and phenotypic data have shown that by inhibiting *soxS*, susceptibility to tetracycline can be restored in multidrug-resistant K. pneumoniae. We were further interested in investigating whether *soxS*-mediated tetracycline susceptibility can be demonstrated in an *in vivo* zebrafish model.

**FIG 5:**
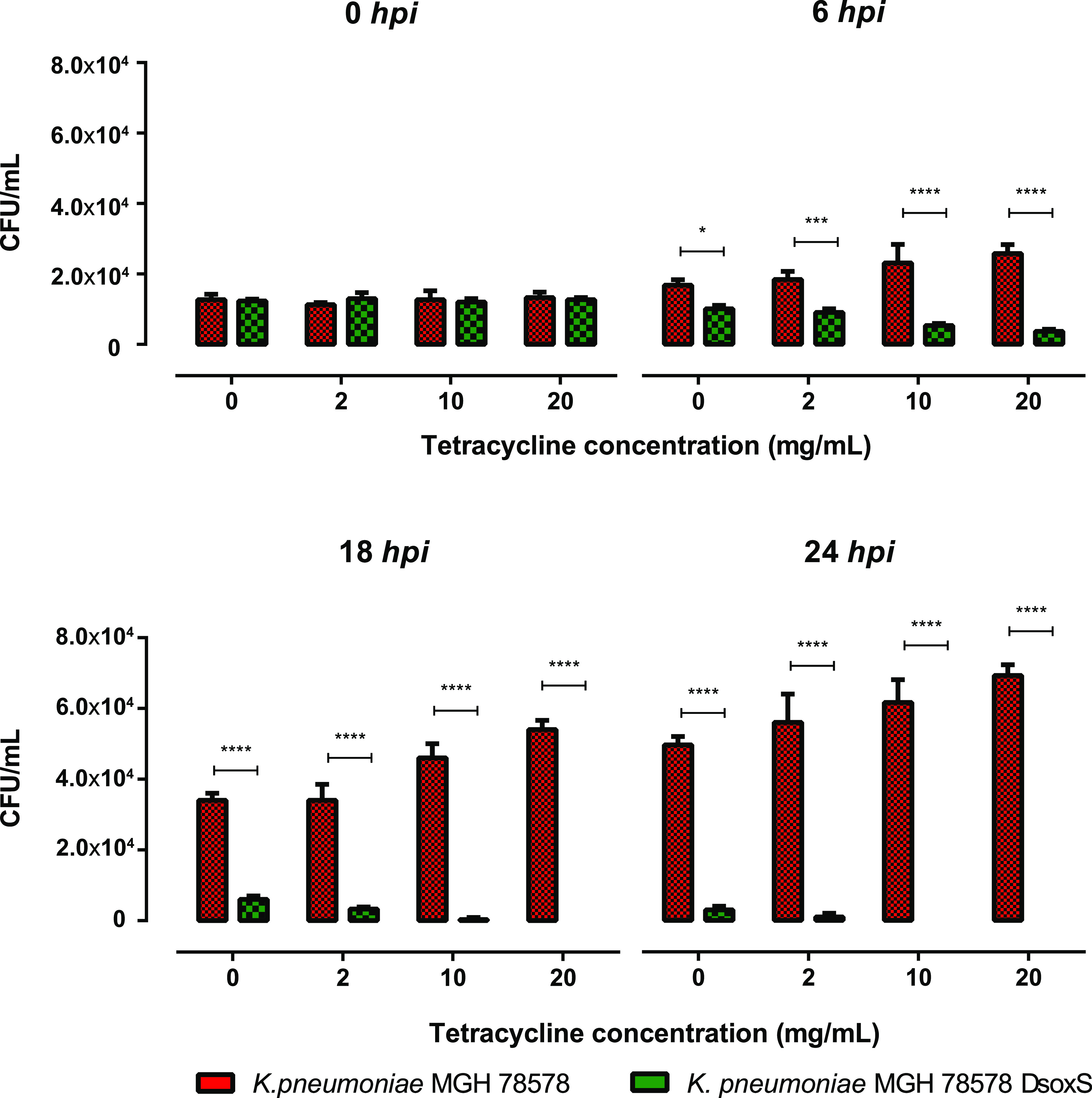
Survival of bacteria in a zebrafish larvae infection model where the larvae are treated with tetracycline. Tetracycline induces ROS generation in the zebrafish larvae. The bar charts show the survival of the different bacterial cultures in the larval blood at 0, 6, 18, and 24 h postinfection and at different concentrations of tetracycline. Error bars represent standard deviations (SD) calculated from 3 independent reads. Significance was determined by two-way ANOVA and Sidak's multiple-comparison test, comparing the different tetracycline concentrations with the control group for each time point.

For this, we treated 4-hpf zebrafish embryos with increasing concentrations of tetracycline. At 48 hpf, K. pneumoniae MGH78578, the K. pneumoniae MGH78578 Δ*soxS* strain, and DPBS were microinjected into the blood circulation. Postinjection, zebrafish larvae were collected at different time points, and again bacterial counts were enumerated (Fig. 5).

It was shown recently that exposure to tetracycline induced ROS production in zebrafish larvae (42). Thus, we hypothesized that the K. pneumoniae MGH78578 Δ*soxS* strain will be impaired in its ability to survive in tetracycline-treated zebrafish larvae due to increased sensitivity to either ROS production in tetracycline-treated larvae or tetracycline alone. It is also possible that healthy zebrafish larvae could clear the K. pneumoniae MGH78578 Δ*soxS* strain from the system due to normal exposure to peroxides synthesized from neutrophils. Confirming our hypothesis, the K. pneumoniae MGH78578 Δ*soxS* strain was completely cleared from the tetracycline-treated zebrafish larvae in 24 h. However, the K. pneumoniae MGH78578 Δ*soxS* strain was cleared even in untreated zebrafish larvae, confirming that the selective advantage was lost in the bacterial mutant, making it susceptible to the immune system of zebrafish larvae (Fig. 5). It should also be noted that the clearance was much more marked in tetracycline-treated larvae, suggesting that the K. pneumoniae MGH78578 Δ*soxS* strain was cleared from the system due to a cumulative effect of both immune system- and tetracycline-induced ROS production. Overall, we show that *soxS* can be used as a genetic target to treat multidrug-resistant K. pneumoniae infections.

### Conclusions.

Apart from elucidating the PQ oxidative stress regulon and the oxidative SoxS regulon, we propose that a combination of tetracycline and an SoxS inhibitor can be used to treat infections associated with MDR K. pneumoniae. A decade ago it was shown that bactericidal antibiotics kill bacteria by unleashing intracellular oxidative stress, a phenomenon independent of the antibacterial action mechanism of antibiotics (24, 43, 44), that harnesses ROS as an effective antibacterial strategy (45). At least in a zebrafish embryo model system, tetracycline is known to induce host oxidative stress (42). Thus, we hypothesized that if tetracycline was used in the presence of an SoxS inhibitor, the overall effect produced was bactericidal, making the tetracycline-SoxS inhibitor combination a potential target for antibiotic drug discovery. Since the target action mechanism is metabolic dysfunction (oxidative stress inhibition), such an approach could work on bacteria regardless of its expressed drug resistance profile.

However, the major limitation of our study is that it is based on a single bacterial strain and its isogenic mutant, and observations recorded in K. pneumoniae MGH78578 may not necessarily apply to other K. pneumoniae strains or other pathogens in a clinical setting. Ours is a proof-of-concept paper, and it will be worthwhile to test if our observations can translate to other isolates of K. pneumoniae and other MDR pathogens, including Salmonella Typhimurium, E. coli, and others. Second, our approach involves oxidative stress inhibition, a mechanism that might not apply if any antibiotic does not employ oxidative stress as a killing mechanism. Nonetheless, since the oxidative stress response is a global bacterial response, the cross-genus application of our predictions, as reported here, may hold some merit. Overall, by providing an interesting target for antibiotic drug discovery, our results address the immediate concern of antimicrobial resistance in pathogens of importance to human health while providing a proof of concept for an approach that requires further experimental investigation to achieve the therapeutic objective.

## MATERIALS AND METHODS

### Bacterial strain.

MDR Klebsiella pneumoniae MGH78578 (ATCC 700721) was isolated from a sputum sample in 1994 and was purchased from the American Type Culture Collection. This strain was selected mainly because it is a multidrug-resistant type strain (46), and its drug resistance profile was recently published (22). Moreover, the efflux pumps present in this strain are well characterized (47, 48). Further, the whole-genome sequence of this strain is available in NCBI (reference sequence NC_009648.1), which was convenient for mapping RNA-seq data. This bacterium was grown in Müeller-Hinton broth (MHB) and Müeller-Hinton agar (MHA) (Sigma, Dublin, Ireland).

### Phenotypic assay (OmniLog).

The comparison of K. pneumoniae MGH78578 WT with the Δ*soxS* mutant was evaluated using the OmniLog (Biolog, Inc., Hayward, CA) phenotypic microarray. Microplates PM1 through PM20, except PM5, were used. These plates contain several carbon, nitrogen, sulfur, and phosphorous substrates, ions, osmolytes, and chemicals at different concentrations and pH (49). Klebsiella pneumoniae MGH78578 WT and Δ*soxS* strains were grown at 37°C on LB agar plates, and several colonies were picked with a sterile cotton swab and suspended in 15 ml IF-0 until a cell density of 42% transmittance (T) was reached (measured using a Biolog turbidimeter). Each 15-ml suspension was then added to 75 ml of physiological solution IF-0 containing dye A, used to inoculate PM plates 1 and 2. PM plates 3 to 8 were inoculated with IF-0 solution containing sodium pyruvate as a carbon source. PM9 to -20 were inoculated with the physiological solution IF-10. One hundred microliters of each mixture was inoculated into each well of the microplates. All PM microplates were incubated at 37°C in an OmniLog reader and monitored for 72 h. Data were analyzed using DuctApe software v 0.17.4 (50). Each strain was analyzed in duplicate. Results are present in Table S2 in the supplemental material.

### Isolation of RNA from oxidatively stressed bacterial cells.

Before RNA isolation, wild-type Klebsiella pneumoniae MGH78578 and the K. pneumoniae MGH78578 Δ*soxS* strain were grown until mid-exponential state (MEP) by following an earlier standardized protocol (22). MEP-grown bacterial cells were treated with paraquat (7.81 μM) for 30 min to generate oxidative stress conditions. RNA was then extracted from both oxidative stressed and MEP-grown (control) cells using the Qiagen RNeasy minikit by following the manufacturer’s guidelines. Contaminating DNA was removed from the RNA sample using the Turbo DNase I kit (Thermo Fischer Scientific). RNA was then quantified using both Qubit RNA broad-range assay and the NanoDrop device.

### Sequencing RNA isolated from oxidatively stressed bacterial cells.

The library preparation and subsequent sequencing were carried out commercially at the Center For Genomic Research, University of Liverpool. The Ribo-Zero rRNA removal kit for bacteria (Illumina, San Diego, CA) was used to carry out the depletion of ribosomal DNA according to the manufacturer’s instructions. Libraries were created using a NEBNext Ultra directional RNA library prep kit (New England BioLabs, Frankfurt, Germany). Pooled libraries were loaded on the cBot (Illumina, San Diego, CA), and cluster generation was performed according to the manufacturer’s instructions. Single-end sequencing using 125-bp read length was performed on an Illumina HiSeq 2500 platform (Illumina HiSeq control software 2.2.38) using an Illumina HiSeq Flow Cell v4 and TruSeq SBS kit v4 (Illumina). Raw sequencing read data were processed using RTA version 1.18.61 and CASAVA 1.8.4 to generate FASTQ files. Genomic cDNA libraries were prepared using the TruSeq stranded total RNA library prep kit (Illumina, San Diego, CA) with Ribo-Zero to deplete rRNA. An average of 1.48 Gbp of raw sequence data was obtained per sample in 125-bp single-end reads.

### Mapping of sequenced reads.

The sequence quality of the RNA-seq reads was analyzed using FastQC (https://www.bioinformatics.babraham.ac.uk/projects/fastqc/). Sequence reads were aligned and mapped against the reference genome of K. pneumoniae MGH78578 (reference sequence NC_009648.1) using Segemehl with default mapping parameters (22, 51), and uniquely mapped reads were used and considered for the differential gene expression computational analysis. Read counts (number of reads that aligned to a specific gene) for each gene were quantified using custom Perl scripts.

### Computational analysis of RNA-seq data.

Computational analysis of RNA-seq data were performed using R (version 3.5.2; https://www.r-project.org/). To calculate the expression level of genes, the raw read counts were normalized using the VOOM function (21) in the limma package (52). More specifically, counts were converted to log_2_ counts per million (log_2_ CPM), quantile normalized, and precision weighted using the VOOM function. A linear model was then fitted to each gene, and empirical Bayes-moderated t-statistics and its corresponding *P* values were used to assess differences in expression (21, 53). To account for multiple comparisons, Benjamini-Hochberg-corrected *P* values were computed. As reads for duplicated coding genes (paralogs) or duplicated small RNAs cannot be mapped unequivocally, these genes appear in the analysis as unmapped. The sequence reads can be visualized in the Integrated Genome Browser (version 9.0.0) (54). The read depth was adjusted with the cDNA library with the lowest number of reads (55). RNA sequencing data were analyzed using the following fold change parameters: highly upregulated (>4), upregulated (2- to 4-fold), no change in expression (0.5- to 2-fold), downregulated (0.25- to 0.5-fold), and highly downregulated (less than 0.25-fold).

### Construction of the K. pneumoniae MGH78578 Δ*soxS* mutant.

A modified λ-Red system was used to construct an in-frame deletion in multidrug-resistant K. pneumoniae MGH78578 (56). Here, three plasmids are employed. The first, plasmid pIJ773, serves as a template to amplify the apramycin resistance gene, *aac(3)IV*, and flanking FLP recombination target (FRT) sites. The second plasmid, pACBSR-Hyg, contains the λ-Red system comprising *beta*, *gam*, and *exo* genes, which are under the control of an arabinose-inducible promoter and facilitate homologous recombination between the knockout cassette and the target locus in the chromosome. The third plasmid, pFLP-Hyg, contains the FLP recombinase, which was used to excise the apramycin selection marker from the chromosome via the FRT sites. The antibiotic apramycin was used to select plasmid pIJ773, while hygromycin was used to select both plasmids pACBSR-Hyg and pFLP-Hyg. K. pneumoniae MGH78578 was susceptible to both apramycin and hygromycin.

Plasmid pACBSR-Hyg was first introduced into wild-type K. pneumoniae MGH78578 by electroporation. An overnight culture of the bacteria was reinoculated into 100 ml Luria-Bertani (LB) broth at 220-rpm aeration and 30°C temperature until an optical density at 600 nm (OD_600_) of 0.6 to 0.8 was reached. The cells were washed twice with 50 ml ice-cold 10% (vol/vol) glycerol and resuspended in the residual glycerol solution after the final wash. A 50-μl aliquot of the dense suspension was then mixed with 200 to 400 ng of plasmid DNA and electroporated at 2,500 mV. Bacterial cells were revived in SOC medium, which was added immediately after electroporation, and K. pneumoniae MGH78578[pACBSR-Hyg] was selected by plating on low-salt LB plates containing hygromycin. The knockout cassette consisted of three distinct regions: *aac(3)IV*, FRT sites that flanked *aac(3)IV*, and 60-bp regions homologous to the *soxS* gene were amplified using PCR from the plasmid pIJ773 as the template. Competent K. pneumoniae MGH78578[pACBSR-Hyg] was prepared by growing the cells in low-salt LB with 1 M l-arabinose and 100 μg/ml hygromycin, with washing in ice-cold 10% (vol/vol) glycerol as described above. The PCR-amplified knockout cassette was electroporated into competent K. pneumoniae MGH78578[pACBSR-Hyg], and cells with successful recombination events were selected by plating in LB with apramycin and incubating overnight at 37°C. K. pneumoniae MGH78578 *soxS*::FRT*-aac(3)IV*-FRT cells were identified and confirmed using PCR primers targeting regions that flanked the *soxS* region. Competent K. pneumoniae MGH78578 *soxS*::FRT*-aac(3)IV*-FRT cells were prepared and electroporated with plasmid pFLP-Hyg to excise the inserted knockout cassette, and the K. pneumoniae MGH78578 Δ*soxS* cells were confirmed using PCR and sequencing. The sequences of all primers used in the experiment are provided in Table S1, worksheet 1 (WS1), in the supplemental material.

### Isolation of RNA for qRT-PCR.

Wild-type K. pneumoniae MGH78578 was grown to mid-exponential phase (MEP) at 37°C in Müeller-Hinton broth. Cells were then treated with paraquat at different concentrations (0, 3.905, 7.81, 200, and 500 μM) or similarly with tetracycline (0, 0.5, 10, 100, and 500 μg/ml) for 30 min, and RNA was then extracted. All assays were run in triplicate. Under all conditions, RNA was extracted using an Qiagen RNeasy minikit by following the manufacturer's guidelines. Any contaminating DNA was removed from the RNA sample using the Turbo DNase I kit (Thermo Fischer Scientific). Purified RNA was subsequently quantified using both Qubit RNA broad-range assay and NanoDrop device.

### Two-step RT-qPCR.

The reverse transcriptase reaction was carried out on RNA purified earlier from K. pneumoniae MGH78578 under the same conditions mentioned earlier. A high-capacity RNA-to-cDNA preparation kit (Sigma) was used by following the manufacturer’s guidelines. A negative control devoid of RT enzyme was also included. qPCR was then performed by following the prime-time gene expression master mix protocol (IDT, Leuven, Belgium) in an Eppendorf Mastercycler RealPlex ep gradient S (Eppendorf, Arlington, United Kingdom) according to the manufacturer’s instructions. Samples were run for 3 biological replicates, each of which had three technical replicates. Data were analyzed using RealPlex software. The relative fold increases in expression levels (changes in threshold cycle [Δ*C_T_*]) were normalized based on the gene expression levels of the housekeeping gene *rpoB* relative to the *soxS* gene. Comparative quantification was carried out using the ΔΔ*C_T_* approach. The sequences of all primers used in the experiment are provided in Table S1, WS1.

### Determination of MBC.

Previously, MIC values for paraquat (PQ), colistin (COL), tetracycline (TET), gentamicin (CN), kanamycin (KM), and cefotaxime (CTX) were determined in triplicate using a 96-well microtiter plate 2-fold broth microdilution method (22). The range of concentrations employed was 1 to 512 μg/ml for all antibiotics, excluding colistin, for which a concentration range of 0.03125 to 16 μg/ml and paraquat of 0.97 to 500 μM was utilized. Overnight LB cultures of K. pneumoniae MGH78578 and the K. pneumoniae MGH78578 Δ*soxS* strain were diluted in sterile phosphate-buffered saline to 10^5^ CFU/ml. A 96-well plate was used to prepare 2-fold serial dilutions of each antibiotic for MHB and MBC determination of K. pneumoniae MGH78578 and the K. pneumoniae MGH78578 Δ*soxS* strain against each compound. A volume of 5 μl of the 10^5^ CFU/ml bacterial culture was then transferred to separate wells containing various concentrations of the compounds to be tested. These plates were then incubated at 37°C for 16 to 18 h according to European Committee on Antimicrobial Susceptibility Testing (EUCAST) 2018 guidelines. Triplicate MBC values for each antibiotic tested were determined using MHB in a 96-well microtiter plate. A steel inoculator was employed to transfer inoculum from the 96-well plate as described above to a fresh 96-well plate containing MHB without any of the antibiotics to be tested. These plates were then incubated at 37°C for 16 to 18 h, following which the MBC values were recorded.

### Electrochemical measurement experiments.

The efflux activity of K. pneumoniae MGH78578 and K. pneumoniae MGH78578 Δ*soxS* cells was assayed measuring the accumulation of tetraphenylphosphonium (TPP^+^) ions. Overnight cultures of K. pneumoniae were grown in Luria-Bertani broth containing 0.5% NaCl, diluted 3:100 in fresh medium, and the incubation was continued until the OD_600_ reached 1.0. The cells were collected by centrifugation at 4°C for 10 min at 3,000 × *g*. The pelleted cells were resuspended in 100 mM sodium phosphate buffer, pH 8, to obtain 1.4 × 10^11^ CFU/ml. Concentrated cell suspensions were kept on ice until used but not longer than 3 h.

Changes in TPP^+^ concentration in the suspensions of thermostated and magnetically stirred cells were monitored using TPP^+^-selective electrodes as previously described (31, 57). Experiments were performed at 37°C in 100 mM sodium phosphate buffer, pH 8, containing 0.1% glucose. The OD_612_ of the cell suspension during measurements was 1.

### Zebrafish line maintenance, infection, and microinjection experiments.

Zebrafish (Danio rerio) used in this study were *wik* lines. Adult fish were kept on a 14-h/10-h light/dark cycle at pH 7.5 and 27°C. Eggs were obtained from natural spawning adult fish, which were set up pairwise in individual breeding tanks. Embryos were raised in petri dishes containing E3 medium (5 mM NaCl, 0.17 mM KCl, 0.33 mM CaCl_2_, 0.33 mM MgSO_4_) supplemented with 0.3 μg/ml methylene blue at 28°C. From 24 hpf, 0.003% 1-phenyl-2-thiourea (PTU) was added to prevent melanin synthesis. The staging of embryos was performed as explained earlier (58).

Microinjections were performed using borosilicate glass microcapillary injection needles (1-mm outer diameter by 0.78-mm inner diameter; 1210332; Science Products) and a PV830 Pneumatic PicoPump (World Precision Instruments). The 48-hpf embryos were manually dechorionated and anesthetized with 200 mg/liter buffered tricine (MS-222; Sigma) before injection. Subsequently, embryos were aligned on an agar plate and injected with 12,000 CFU in a 1- to 4-μl volume of a bacterial suspension in DPBS directly into the blood circulation (caudal vein, *n* = three sets of 10). Before injection, the volume of the injection suspension was adjusted by injecting a droplet into mineral oil and measuring its approximate diameter over a micrometer scale bar. The number of CFU injected was determined by injection of bacterial suspension into a DPBS droplet on the agar plate. Following injections, injected embryos were allowed to recover in a petri dish with fresh E3 medium for 15 min. To monitor infection kinetics and for survival assays, embryos were transferred into 24-well plates (one embryo per well) containing 1 ml E3 medium per well, incubated at 28°C, and observed for survival under a stereomicroscope twice a day. For survival assays after infection, the number of dead larvae was determined visually based on the absence of a heartbeat. Kaplan-Meier survival analysis and statistics for experiments with zebrafish were done with GraphPad Prism 7 (GraphPad Software). Experiments were performed in triplicate.

### Zebrafish tetracycline exposure experiments.

Wild-type (*wik* strain) zebrafish embryos were used for this study. Four-hpf embryos from three different pairs were examined under stereomicroscope for normal development, and embryos that had reached the blastula stage were selected for the following experiments. Embryos (*n* = three sets of 10 for each tetracycline concentration used) were randomly transferred into each well of 24-well plates containing 2 ml of E3 medium. A series of tetracycline concentrations (0, 2, 10, and 20 μg/liter) were applied and maintained until 48 hpf at 28°C. The solutions were changed once every 24 h. At 48 hpf, embryos were manually dechorionated, anesthetized, and microinjected directly into the blood circulation as mentioned above. Following injections, injected embryos were allowed to recover in a petri dish with fresh E3 medium for 15 min and subsequently transferred into each well of 24-well plates containing fresh E3 medium and the respective concentration of tetracycline. Embryos or larvae were collected at each time point (0, 6, 18, and 24 hpi) and independently treated for bacterial enumeration. Significance was determined with GraphPad Prism 7 (GraphPad Software) by applying a two-way analysis of variance (ANOVA) and Sidak's multiple-comparison test, comparing the different tetracycline concentrations with the control group for each time point.

### Ethics statement.

This study was performed by following the principles and recommendations of the “Ordinance on laboratory animal husbandry, the production of genetically modified animals and the methods of animal experimentation; Animal Experimentation Ordinance” (SR 455.163, 12 April 2010), Swiss Federal Food Safety and Veterinary Office (FSVO/BLV). The maximum age reached by the embryos during experimentation was 5 days postfertilization (dpf), for which no license is required from the cantonal veterinary office in Switzerland, since such embryos will not have reached the free-feeding stage. Husbandry and breeding of the adult zebrafish were performed under the supervision of Stephan Neuhauss, Institute for Molecular Life Sciences, University of Zurich, Zurich, Switzerland (Cantonal Veterinary Office of Zurich, husbandry license no. 150). All animal protocols used were in compliance with internationally recognized standards as well as with Swiss legal ethical guidelines for the use of fish in biomedical research.

### Data availability.

All the RNA sequence data generated in the study were deposited in the National Center for Biotechnological Information–Gene Expression Omnibus and are available under the accession number GSE146844. Postanalysis, the differential expression levels of all the genes are given in Table S1 with three worksheets, WS-1, WS-2, and WS-3.
