# Supplementary figures and images for: Analysis of the Oxidative Stress Regulon Identifies soxS as a Genetic Target for Resistance Reversal in Multidrug-Resistant Klebsiella pneumoniae
Source: mBio. 2021 Jun 8;12(3):e00867-21. doi: 10.1128/mBio.00867-21 (PMC8262902; doi:10.1128/mBio.00867-21)

Supplementary figures

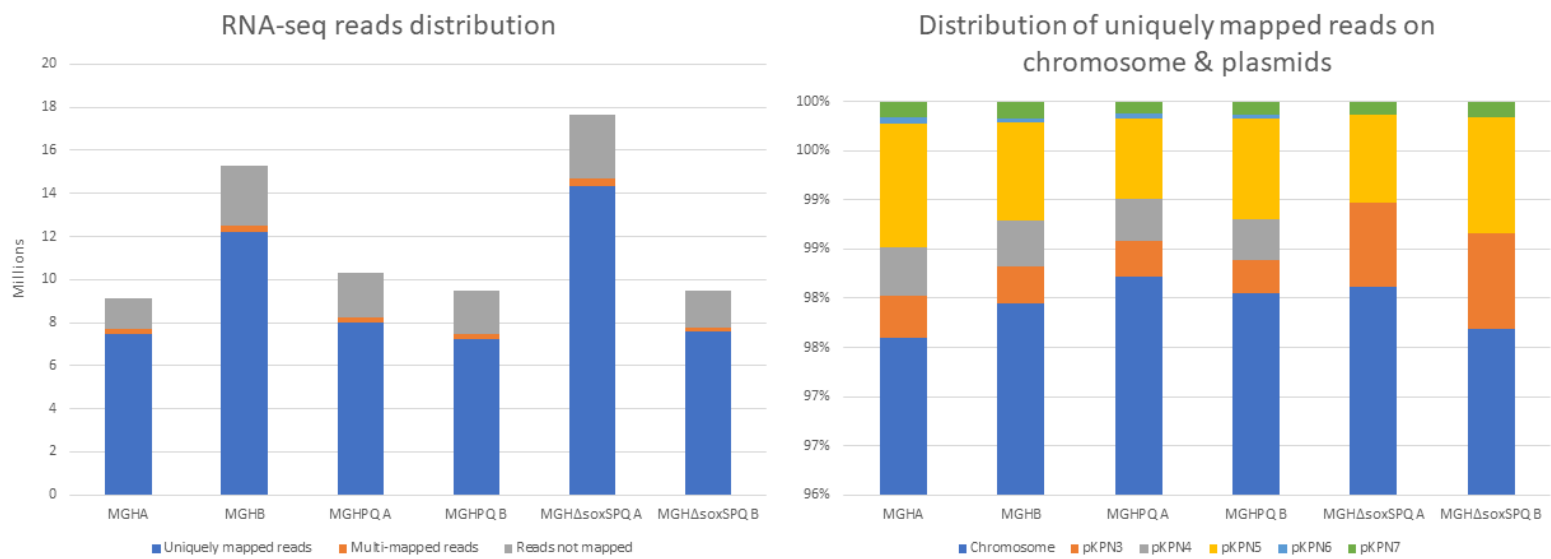

**Figure S1.** Distribution of RNA-seq reads in each individual dataset.

Supplement: FIG S1 [file mbio.00867-21-sf001.pdf]
